# GATEKEEPER’s Strategy for the Multinational Large-Scale Piloting of an eHealth Platform: Tutorial on How to Identify Relevant Settings and Use Cases

**DOI:** 10.2196/42187

**Published:** 2023-06-28

**Authors:** Jordi de Batlle, Ivan D Benítez, Anna Moncusí-Moix, Odysseas Androutsos, Rosana Angles Barbastro, Alessio Antonini, Eunate Arana, Maria Fernanda Cabrera-Umpierrez, Gloria Cea, George Ε Dafoulas, Frans Folkvord, Ane Fullaondo, Francesco Giuliani, Hsiao-Ling Huang, Pasquale F Innominato, Przemyslaw Kardas, Vivian W Q Lou, Yannis Manios, Maria Matsangidou, Franco Mercalli, Mounir Mokhtari, Silvio Pagliara, Julia Schellong, Lisa Stieler, Konstantinos Votis, Paula Currás, Maria Teresa Arredondo, Jorge Posada, Sergio Guillén, Leandro Pecchia, Ferran Barbé, Gerard Torres, Giuseppe Fico, the GATEKEEPER project

**Affiliations:** 1 Group of Translational Research in Respiratory Medicine Institut de Recerca Biomedica de Lleida Hospital Universitari Arnau de Vilanova-Santa Maria Lleida Spain; 2 Center for Biomedical Network Research in Respiratory Diseases Madrid Spain; 3 Lab of Clinical Nutrition and Dietetics, Department of Nutrition and Dietetics School of Physical Education, Sport Science and Dietetics University of Thessaly Trikala Greece; 4 Unidad de Innovación Servicio Aragonés de Salud Hospital de Barbastro Barbastro Spain; 5 Knowledge Media Institute The Open University Milton Keynes United Kingdom; 6 Biocruces Bizkaia Health Research Institute Osakidetza Barakaldo Spain; 7 Life Supporting Technologies Escuela Técnica Superior de Ingenieros de Telecomunicaciones Universidad Politécnica de Madrid Madrid Spain; 8 E-health Department Digital Cities of Central Greece Trikala Greece; 9 Department of Endocrinology and Metabolic Diseases Faculty of Medicine University of Thessaly Larisa Greece; 10 PredictBy Barcelona Spain; 11 Tilburg School of Humanities and Digital Sciences Tilburg Netherlands; 12 Kronikgune Institute for Health Services Research Barakaldo Spain; 13 Innovation and Research Department Fondazione Casa Sollievo della Sofferenza Research Hospital San Giovanni Rotondo Italy; 14 Department of Healthcare Management Office of International and Cross-Strait Affairs Yuanpei University of Medical Technology Hsinchu Taiwan; 15 Oncology Department Ysbyty Gwynedd Betsi Cadwaladr University Health Board Bangor United Kingdom; 16 Warwick Medical School & Cancer Research Centre University of Warwick Coventry United Kingdom; 17 Faculty of Medicine Paris-Saclay University Villejuif France; 18 Medication Adherence Research Centre Department of Family Medicine Medical University of Lodz Lodz Poland; 19 Department of Social Work and Social Administration Sau Po Center on Ageing The University of Hong Kong Hong Kong China; 20 Department of Nutrition & Dietetics School of Health Science & Education Harokopio University Athens Greece; 21 Institute of Agri-food and Life Sciences Hellenic Mediterranean University Research Centre Heraklion Greece; 22 Cyens Centre of Excellence Nicosia Cyprus; 23 MultiMed Engineers srl Parma Italy; 24 Scientific Direction Institut Mines-Telecom Paris France; 25 National University of Singapore Singapore Singapore; 26 School of Engineering University of Warwick Coventry United Kingdom; 27 Department of Psychotherapy and Psychosomatic Medicine Faculty of Medicine Technische Universität Dresden Dresden Germany; 28 Information Technologies Institute Centre for Research and Technology Hellas Thessaloniki Greece; 29 Innova & European Projects Office Integrated Health Solutions Medtronic Ibérica S.A. Madrid Spain; 30 Mysphera S.L. Paterna Spain; 31 See Acknowledgments

**Keywords:** big data, chronic diseases, eHealth, healthy aging, integrated care, large-scale pilots

## Abstract

**Background:**

The World Health Organization’s strategy toward healthy aging fosters person-centered integrated care sustained by eHealth systems. However, there is a need for standardized frameworks or platforms accommodating and interconnecting multiple of these systems while ensuring secure, relevant, fair, trust-based data sharing and use. The H2020 project GATEKEEPER aims to implement and test an open-source, European, standard-based, interoperable, and secure framework serving broad populations of aging citizens with heterogeneous health needs.

**Objective:**

We aim to describe the rationale for the selection of an optimal group of settings for the multinational large-scale piloting of the GATEKEEPER platform.

**Methods:**

The selection of implementation sites and reference use cases (RUCs) was based on the adoption of a double stratification pyramid reflecting the overall health of target populations and the intensity of proposed interventions; the identification of a principles guiding implementation site selection; and the elaboration of guidelines for RUC selection, ensuring clinical relevance and scientific excellence while covering the whole spectrum of citizen complexities and intervention intensities.

**Results:**

Seven European countries were selected, covering Europe’s geographical and socioeconomic heterogeneity: Cyprus, Germany, Greece, Italy, Poland, Spain, and the United Kingdom. These were complemented by the following 3 Asian pilots: Hong Kong, Singapore, and Taiwan. Implementation sites consisted of local ecosystems, including health care organizations and partners from industry, civil society, academia, and government, prioritizing the highly rated European Innovation Partnership on Active and Healthy Aging reference sites. RUCs covered the whole spectrum of chronic diseases, citizen complexities, and intervention intensities while privileging clinical relevance and scientific rigor. These included lifestyle-related early detection and interventions, using artificial intelligence–based digital coaches to promote healthy lifestyle and delay the onset or worsening of chronic diseases in healthy citizens; chronic obstructive pulmonary disease and heart failure decompensations management, proposing integrated care management based on advanced wearable monitoring and machine learning (ML) to predict decompensations; management of glycemic status in diabetes mellitus, based on beat to beat monitoring and short-term ML-based prediction of glycemic dynamics; treatment decision support systems for Parkinson disease, continuously monitoring motor and nonmotor complications to trigger enhanced treatment strategies; primary and secondary stroke prevention, using a coaching app and educational simulations with virtual and augmented reality; management of multimorbid older patients or patients with cancer, exploring novel chronic care models based on digital coaching, and advanced monitoring and ML; high blood pressure management, with ML-based predictions based on different intensities of monitoring through self-managed apps; and COVID-19 management, with integrated management tools limiting physical contact among actors.

**Conclusions:**

This paper provides a methodology for selecting adequate settings for the large-scale piloting of eHealth frameworks and exemplifies with the decisions taken in GATEKEEPER the current views of the WHO and European Commission while moving forward toward a European Data Space.

## Introduction

Worldwide socioeconomic improvements have prolonged and will further prolong life expectancy, progressively shifting the structure of population pyramids toward aging populations [1]. Therefore, healthy aging, defined by the World Health Organization (WHO) as “the process of developing and maintaining the functional ability that enables well-being in older age,” has become one of the greatest challenges of our society [2]. The United Nations and the WHO have established 2020-2030 as the Decade of Healthy Aging [3], highlighting the following four key action areas: (1) changing the perception of age and aging, (2) fostering older people’s abilities, (3) delivering person-centered integrated care and services, and (4) providing access to long-term care. Moreover, they have stated the need to innovate in the collection, sharing, analysis, and use of healthy-aging–related data.

eHealth [4] must be a cornerstone in achieving such goals, as it can foster accessibility, interoperability, security, and efficiency while making the citizens play an active role in their own management. As recently reviewed by Beks et al [5], eHealth-delivered community health programs can successfully tackle the day-to-day management of prevalent chronic diseases. Similarly, eHealth can be and is being effectively used in the promotion of healthy aging [6]. Nevertheless, the large-scale implementation of eHealth poses many challenges in the fields of implementation, ethics and legal aspects, data management and protection, technical development, and end user engagement [7]. In this regard, federated implementations could provide a good balance between the complexity of fully centralized systems and the poor interoperability of decentralized systems [7]. Unfortunately, self-management platform–like interventions and platforms accommodating multiple such interventions are yet very scarce [8], and there are only a few examples of how to implement or test them adequately [9].

The European Horizon 2020 project GATEKEEPER (Smart living homes—whole interventions demonstrator for people at health and social risks) is a European multicentric large-scale pilot on smart living environments [10]. In line with the principles of the Decade of Healthy Aging [3], GATEKEEPER aims to create a platform connecting health care providers, enterprises, and older citizens and their communities, to originate an open, trust-based arena for matching ideas, technologies, user needs, and processes, ultimately ensuring healthier independent lives for the aging populations. The GATEKEEPER platform will be embodied in an open-source, European, standard-based, interoperable, and secure framework available to all developers for creating combined digital solutions for personalized and precise early detection and intervention approaches. Finally, as a large-scale pilot, the GATEKEEPER platform will need to be implemented and tested in different European regions, enrolling tens of thousands of older citizens to assess its functionality and sustainability.

The GATEKEEPER project differs from most eHealth-related projects because of its broader scope. GATEKEEPER aims toward implementing a platform serving broad populations of aging citizens with heterogeneous needs, rather than disease-specific eHealth systems or settings targeting well-defined subgroups of citizens with homogeneous characteristics. Therefore, the definition of the most appropriate group of settings where the platform should be tested represents a challenge on its own. This paper describes the rationale for the selection of the group of settings used to test the GATEKEEPER platform, and presents the different implementation sites and reference use cases (RUCs), to provide an overview of the clinical and scientific meaningfulness of this platform.

## Methods

**Figure 1:**
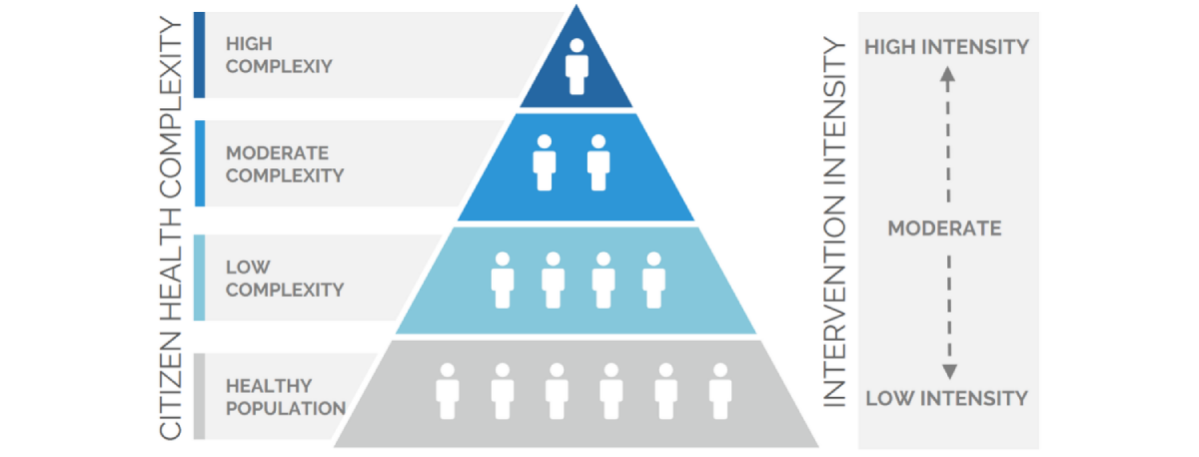
GATEKEEPER double stratification pyramid: citizen health complexity and intervention intensity.

Testing a platform capable of accommodating any number and type of eHealth solutions for broad study populations of older citizens with diverse needs requires being systematic. To do so, we adopted a double stratification pyramid reflecting at the same time the overall health of the target study population for a given intervention as well as the intensity of the proposed intervention (Figure 1). The first pyramid, namely “citizen complexity,” focused on the target population’s health risk based on health complexity, and is based on the Kaiser Permanente risk stratification pyramid [11]. Such a pyramid classifies a given study population into one of four strata: (1) a healthy study population, (2) a study population with subjects with risk factors or low complexity patients, (3) a study population with moderate complexity chronic patients, and (4) a study population with high complexity chronic patients. The classification of individual subjects according to their health complexity has been widely used [12]. However, in GATEKEEPER, we aimed at applying such tools at the study population level rather than at the individual level. This means that we classified our target study populations according to their collective health risk. The second pyramid, namely “intervention intensity,” classified eHealth interventions according to their intensity, where the intensity was related to characteristics, such as the need for commercial-grade or medical-grade devices, the need to involve health care professionals or the amount of data being collected (both in terms of a number of parameters and frequency of collection). This second pyramid had 3 strata corresponding to low, moderate, and high intensity, and is a much simpler tool than the WHO Classification of Digital Health Interventions [13]. The need for a double stratification pyramid was raised from the fact that, although most high-intensity interventions are to be delivered to high-complexity citizens (just as low-intensity interventions are usually provided to healthy or low-risk citizens), there is the possibility of having intensity-complexity mismatches better fitting-specific scenarios and aims.

Once the GATEKEEPER double stratification pyramid was established, the next step involved the selection of deployment sites and the identification of relevant RUCs to be implemented. Two considerations were taken into account for the selection of implementation sites. First, it was important to test the system in as many different countries as possible, especially given the differences in regulations, health care systems’ characteristics, and study population needs. In this regard, we plotted candidate countries by latitude and nominal Gross Domestic Product per capita (US dollar) from the International Monetary Fund’s 2021 World Economic Outlook Database [14], to ensure a heterogeneous sample. Second, it was required that each implementation region consisted of an ecosystem composed of health care organizations and partners from industry, civil society, academia, and government authorities all committed to implementing comprehensive, innovation-based approaches to healthy aging. In this sense, the highly rated European Innovation Partnership on Active and Healthy Aging (EIP on AHA) reference sites [15] were prioritized. Regarding the selection of RUCs to be implemented, there were also 2 key considerations. First, we wanted our site-specific pilots to cover the whole spectrum of citizen complexities and intervention intensities, thus the definitions of the RUCs needed to be broad enough to accommodate all possible combinations. This implied, for instance, not neglecting high-intensity interventions for low-complexity patients and low-intensity interventions for high-complexity patients. Second, we wanted each RUC to be scientifically sound and clinically relevant, thus providing solutions to different substantial and pertinent challenges of healthy aging and being able to generate high-value real-world data.

## Results

### Implementation Sites

**Figure 2:**
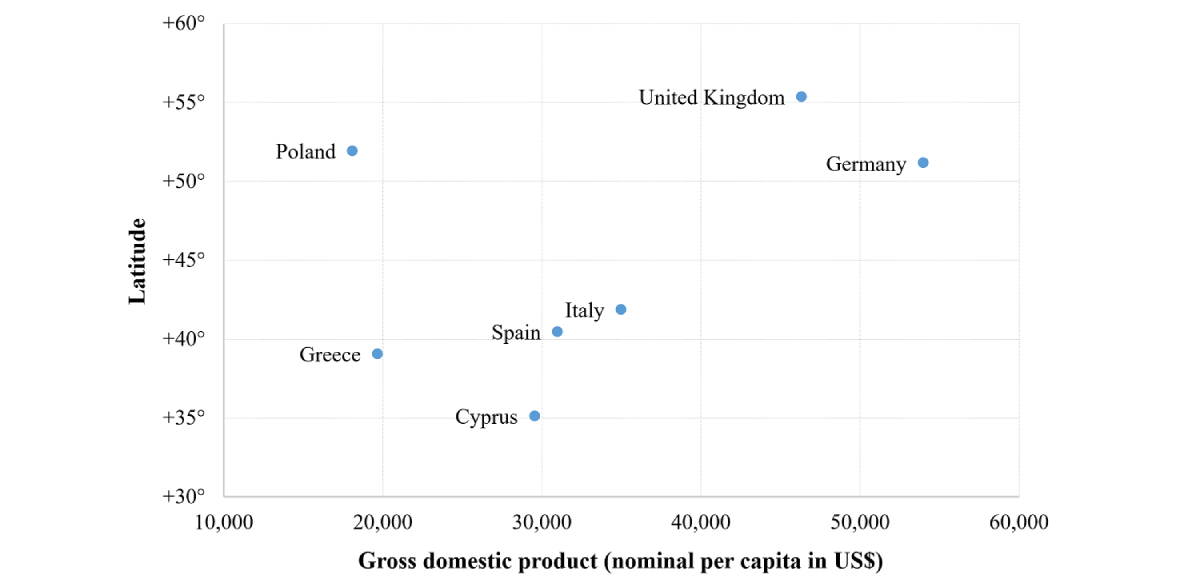
GATEKEEPER's implementation countries by Latitude and nominal Gross Domestic Product per capita (US dollar) according to 2021 data for members of the International Monetary Fund.

Seven European countries were selected by trying to cover the geographical (north-south and east-west) and socioeconomic (spectrum of gross domestic product) heterogeneity of Europe: Cyprus, Germany, Greece, Italy, Poland, Spain, and the United Kingdom (Figure 2). Within these countries, implementation regions or sites were identified based on the high standards that were required. Each implementation site consisted of an ecosystem of key partners trying to include, or be in close contact with, health care organizations, industry, civil society, academia, and government authorities; all committed to implementing comprehensive, innovation-based approaches to healthy aging, choosing highly rated EIP on AHA reference sites when possible (Table 1). Additionally, 3 implementation sites outside of Europe have been added to test the GATEKEEPER platform in completely different settings in terms of sociodemographic, health care–related, and regulatory-related characteristics: Hong Kong (China), Singapore, and Taiwan (Table 1).

**Table 1:** Summary of GATEKEEPER’s implementation sites.

Country and region or site		Key partners
**Cyprus**		
	Cyprus	• Rehabilitation Center for patients with Alzheimer • Cyprus Association of Cancer Patients and Friends
**Germany**		
	Saxony	• Technische Universität Dresden • Carus Consilium Sachsen
**Greece**		
	Attica	• Harokopio University, Athens • Centre for Research and Technology Hellas, Thessaloniki • University of Ioannina
	Central Greece	• Intermunicipal Development Company Digital Cities of Central Greece SA • University of Patras • Centre for Research and Technology Hellas, Thessaloniki • University of Ioannina
**Italy**		
	Puglia	• Regione Puglia • Fondazione Politecnico Milano • MultiMed Engineers srls • Fondazione Casa Sollievo della Sofferenza
**Poland**		
	Lodz	• Medical University of Lodz
**Spain**		
	Aragon	• Servicio Aragonés de Salud • Universidad Politécnica de Madrid
	Basque Country	• Osakidetza • Kronikgune Institute for Health Services Research • Ibermatica
**United Kingdom**		
	Milton-Keynes (England)	• Open University • Samsung UK • SPIROCCO
	Bangor (Wales)	• Bangor Cancer Unit, Ysbyty Gwynedd, BCUHB, NHS Wales • Open University • Samsung UK • University of Ioannina
**China**		
	Hong Kong	• University of Hong Kong
**Singapore**		
	Singapore	• Institut Mines-Telecom, France • National University of Singapore
**Taiwan**		
	Taiwan	• Yuanpei University of Medical Technology • ASUS

### Framework of RUCs

Foremost, RUCs should act as demonstrators of the clinical and scientific value of the GATEKEEPER platform. All of the Project’s RUCs were built upon existing expertise and resources matured in previous projects, such as ACTIVAGE [16]. The central actors in all GATEKEEPER RUCs are older citizens, who are to be empowered through apps for healthy lifestyle promotion or sets of monitoring tools, aiming to improve health-related quality of life. The generation of substantial amounts of high-quality data ready to be analyzed and used as a key common feature across all the RUCs. The main characteristics of GATEKEEPER’s RUCs are summarized below, including insight into the target study populations, main objectives, and potential key enabling technologies (KETs; Table 2).

**Table 2:** Main characteristics of GATEKEEPER’s reference use cases (RUCs).

RUC	Target study population	Main objective	Potential KETs^a^	Implementation sites
RUC1: Lifestyle-related early detection and interventions	• Older healthy citizens • Older citizens at risk of chronic diseases or mental impairment	• Health promotion • Early detection	• Self-management app, enabling healthy lifestyle promotion or digital coach • Monitoring devices (physical activity, blood pressure, etc)	• Attica (Greece) • Central Greece (Greece) • Aragon (Spain) • Basque Country (Spain) • Hong Kong (China) • Milton Keynes (United Kingdom) • Lodz (Poland) • Puglia (Italy) • Saxony (Germany) • Singapore • Taiwan
RUC2: COPD^b^ exacerbations management	• Older patients with COPD participating in regional programs	• Remote monitoring of COPD signs and symptoms • Predictive modeling for early decompensation detection • Early intervention	• Monitoring devices (physical activity, blood pressure, etc) • Self-management app, enabling healthy lifestyle promotion, or digital coach • Professionals platform or dashboard	• Aragon (Spain) • Puglia (Italy) • Singapore
RUC3: Diabetes, predictive modeling of glycemic status	• Older patients with diabetes • Older citizens with poor metabolic control and associated comorbidities	• Remote continuous monitoring of glycemic status • Predictive modeling for early decompensation detection • Early intervention • Healthy lifestyle promotion	• Continuous glucose monitoring system • Self-management app, enabling healthy lifestyle promotion or digital coach • Professionals platform or dashboard	• Basque Country (Spain) • Central Greece (Greece) • Puglia (Italy) • Singapore
RUC4: PD^c^ treatment decision support system	• Older patients with moderate or advanced PD (moderate motor symptoms, limitations in Activities of daily living, and good response to conventional pharmacologic therapy)	• Improved self-management • Remote monitoring of motor and nonmotor complications • Predictive modeling of indication of second line therapies • Early intervention	• Holter-type device measuring PD symptoms • Electronic pill boxes • Monitoring devices (physical activity, blood pressure, etc) • Cognitive, behavioral, and mood screening app • Professionals platform or dashboard	• Basque Country (Spain)
RUC5: Predicting readmissions and decompensations in HF^d^	• Older patients with HF participating in regional programs	• Remote monitoring of HF signs and symptoms • Predictive modeling for early decompensation detection • Early intervention	• Monitoring devices (physical activity, blood pressure, etc) • Self-management app, enabling healthy lifestyle promotion or digital coach • Professionals platform or dashboard	• Aragon (Spain) • Puglia (Italy)
RUC6: Primary and secondary stroke prevention	• Older patients at high risk of stroke • Older patients with a stroke	• Health education and empowerment for patients and carers • Early identification of stroke signs • Early intervention • Healthy lifestyle promotion	• Advanced educational tools (including virtual or augmented reality) • Monitoring devices (physical activity, blood pressure, etc) • Self-management app, enabling healthy lifestyle promotion and cognitive or behavioral status screening or digital coach • Professionals platform or dashboard	• Basque Country (Spain) • Hong Kong (China)
RUC7: Multichronic older patient management including polymedication	• Older patients with multiple chronic diseases • Older patients with cancer or cancer survivors • Older patients with suspected or at risk of mental-health impairment	• Health promotion • Implementation or enhancement of CCM^e^. • Remote monitoring of signs and symptoms • Predictive modeling for early decompensation detection (physical, mental, and social) • Early intervention	• Self-management app, enabling healthy lifestyle promotion or digital coach • Electronic pill-boxes • Monitoring devices (physical activity, blood pressure...) • Professionals platform or dashboard	• Aragon (Spain) • Basque Country (Spain) • Cyprus • Milton Keynes and Bangor (United Kingdom) • Lodz (Poland) • Puglia (Italy) • Saxony (Germany)
RUC8: eHealth solutions for the management of high blood pressure	• Older citizens with HBP^f^ participating in regional programs	• Health promotion • Remote monitoring of blood pressure • Predictive modeling for early decompensation detection • Early intervention	• Self-management app, enabling healthy lifestyle promotion or digital coach • Monitoring devices (physical activity, blood pressure, etc) • Professionals platform or dashboard	• Puglia (Italy) • Hong Kong (China)
RUC9: eHealth solutions for the management of COVID-19	• Older citizens affected with mild to moderate COVID-19 (not requiring hospitalization or already discharged) • Older healthy citizens affected by pandemic-management measures	• Remote monitoring of COVID-19 signs • Management of self-isolation and self-referral for support • Predictive modeling for early decompensation detection • Early intervention • Population-based management of COVID-19 • Population-based management of the social impact of COVID-19	• Monitoring devices (physical activity, blood pressure, etc) • Self-management app, enabling self-monitoring, medical education and health literacy, and communication with health or social care professionals or community-based support organizations • Professionals platform or dashboard (for health or social or community carers)	• Aragon (Spain) • Milton Keynes (United Kingdom)

^a^KET: key enabling technology.

^b^COPD: chronic obstructive pulmonary disease.

^c^PD: Parkinson disease.

^d^HF: heart failure.

^e^CCM: chronic care models.

^f^HBP: high blood pressure.

#### RUC1: Lifestyle-Related Early Detection and Interventions

The main purpose of RUC1 is the promotion of healthy lifestyles among healthy older people to prevent or delay the onset and worsening of controlled common chronic diseases, such as obesity or hypertension. RUC1 will be based on timely interventions provided by artificial intelligence–based digital coaches through natural language processing techniques, patient-generated longitudinal multidimensional data analyses, structured conversations, consultation, and education. Big data analytics techniques will be exploited to address risk stratification and early detection, based on lifestyle analysis, including pattern recognition for the improvement of public health surveillance [17,18] and for the early detection of mental health impairments or cognitive decline [19] and frailty [20]; data mining for inductive reasoning and exploratory data analysis [21]; and cluster analysis for the identification of high-risk groups among elder citizens [22,23].

#### RUC2: Chronic Obstructive Pulmonary Disease Exacerbations Management

Chronic obstructive pulmonary disease has a huge burden worldwide, accounting for 3.3 million deaths and 74.4 million disability-adjusted life-years (DALYs) in 2019 [24]. RUC2 proposes novel integrated care management for patients with chronic obstructive pulmonary disease, aiming at early detection of the appearance of exacerbations, avoidance of transitions to higher complexity stadia, and preservation of functional status [25]. At its core, machine learning methods based on Dynamic Bayesian Networks, suitable for modeling knowledge and handling time series data, could be used to implement systems that predict exacerbations and avoid hospitalizations. These systems will be built on top of advanced wearable monitoring KETs capable of generating time series data for potentially key variables, such as blood pressure, pulse oximetry, electrocardiogram, respiration rate, skin temperature, or locomotor activity.

#### RUC3: Diabetes Mellitus, Predictive Modeling of Glycemic Status

Diabetes mellitus (DM) is the leading cause of blindness, end-stage renal failure, nontraumatic limb amputations, and cardiovascular morbidity and mortality and has an immense burden worldwide [26]. RUC3 aims to establish patterns that detect related and preventable factors of alterations in metabolic control for citizens with type 2 DM. Measures related to lifestyle and metabolic and medication control will be considered to enable tertiary prevention (decrease in comorbidity, exacerbations, and decompensations). In this sense, and considering that short-term prediction of glycemic dynamics is essential to improve DM self-management, a personalized, adaptive, real-time data-driven computational solution based on data federation in the health care space, identifying the different modes of the underlying glucose metabolism will be provided, aiming to prevent hypoglycemic events. A set of monitoring devices will collect clinical data at home such as, bio- and physiological signals (ie, blood glucose concentration data or continuous glucose monitoring data, galvanic skin response, and heart rate variability) combining them with adaptive machine learning regression models to trigger promptly alarms and signs, allowing for personalized, precise, and timely interventions. Additionally, a novel prognostic index for disease control in people with type 2 DM will be built upon data gathered both from blood samples and from smartwatch-generated data linked to lifestyles (number of steps, calorie consumption, heart rate, and sleep).

#### RUC4: Parkinson Disease Treatment Decision Support System

Parkinson disease (PD) is a long-term degenerative disorder of the central nervous system that mainly affects the motor system [27]. It is a common condition, with approximately 6.1 million people affected worldwide in 2016 [28]. RUC4 will target patients with PD with early motor complications, commonly motor complications related to PD treatment, such as unpredictable motor fluctuations and dyskinesia. The main objective of RUC4 will be to the improve quality and efficiency of clinical care for patients with advanced PD by using continuous monitoring of motor and nonmotor complications to identify when the disease has progressed to a point requiring a change in medical therapy. GATEKEEPER KETs, such as wearable sensors to continuously or periodically measure motor symptoms (depending on disease severity) and digital applications, which can be used to detect nonmotor symptoms could be used to record data on the patient’s electronic health records, accessible in the GATEKEEPER Health care Space, and feed machine learning models alerting clinicians when the patient’s current treatment plan is not optimal anymore and will derive suggestions on how to improve it.

#### RUC5: Predicting Readmissions and Decompensations in Heart Failure

Heart failure (HF) is a common disease, being the second leading cause of global DALYs loss in 2019 [29]. RUC5 proposes novel integrated care management for patients with HF, aiming at early detection of the appearance of decompensations, avoidance of transitions to higher complexity strata, and preservation of functional status. Telemonitoring services and machine learning with Dynamic Bayes Networks will be harnessed to implement an advanced model for predicting HF decompensations, considering comorbidities. Building on the experience of the Multisensor Monitoring in Congestive Heart Failure trial [30], GATEKEEPER Health care Space apps will allow to explore which other longitudinal data measured by GATEKEEPER Consumer Space “things” (ie, bio-impedance, heart rate, respiratory rate and volume, or physical activity duration and intensity) can be used for predicting decompensations.

#### RUC6: Primary and Secondary Stroke Prevention

Stroke is a common disease, being the third leading cause of global DALYs loss in 2019 [29]. RUC6 will target patients who had a cerebrovascular accident, and their carers, with the aim of delivering empowerment programs focused on the early detection of symptoms, improving lifestyle, and adherence to pharmacological and nonpharmacological treatments. Early detection will be tackled from 2 different fronts. On one hand, patients and carers will be trained in the early detection of stroke symptoms (using coaching apps or simulation of real cases with web-based and augmented reality), and thus be able to react faster during an episode of reinfarction. In contrast, a set of monitoring tools providing measures, such as physical activity and adherence to treatment will try to establish the risks associated with a new stroke. These monitoring tools will be wrapped up in a patient’s app that can also screen for cognitive, behavioral, and mood status, and provide web-based coaching features fostering the promotion of self-management (ie, the WeRISE App [31]).

#### RUC7: Multichronic Older Patient Management Including Polymedication

RUC7 targets chronic older patients with variable complexity according to GATEKEEPER’s risk stratification pyramid. The EIP on AHA blueprint “personas” can provide good examples of potential participant profiles [32]. RUC7 proposes the implementation of novel chronic care models (CCM) for multimorbid subjects, or the enhancement of already existing CCM, using the possibilities of the different GATEKEEPER Spaces. Several sensing technologies, available in the GATEKEEPER Things Catalogue, can be leveraged and integrated into an unobtrusive mobile data collection platform (ie, based on smartphones and smart trackers), able to monitor the multiple parameters required in CCM for multimorbid subjects. Through the GATEKEEPER Health care Space, data could be shared with clinical professionals in charge of managing the CCMs, to adjust individual care plans accordingly. Through the GATEKEEPER Ecosystem Transaction Space, robotics KETs (from very simple pill dispensers to more complex social robots [33]) could be integrated with digital coaching systems to assist polymedicated patients (ie, in particular for patients who are concurrently affected by cognitive impairments).

#### RUC8: eHealth Solutions for the Management of High Blood Pressure

Hypertension or high blood pressure (HBP), is a serious medical condition with an estimated worldwide prevalence of 31% of adults (1.4 billion) that significantly increases the risk of heart, brain, kidney, and other diseases [34]. RUC8 proposes novel integrated care management for patients with HBP, aiming for the monitoring of blood pressure and early detection of health complications (ie, heart problems and stroke). Telemonitoring services of different intensities will be harnessed to implement periodic or continuous monitoring. The comprehensive analysis of the generated data flow, channeled through the GATEKEEPER systems, will ultimately provide predictions anticipating the appearance of decompensations and triggering alerts and alarms. The intensity of telemonitoring will range from high-intensity monitoring devices in the GATEKEEPER Consumer Space “things” such as wrist monitors, including measurements of other vitals that are important for the close follow-up of patients with HBP, to low-intensity health promotion apps using Optical Character Recognition technology to capture blood pressure data with family-based management features.

#### RUC9: eHealth Solutions for the Management of COVID-19

As of January 2023, more than 650 million COVID-19 cases and 6.7 million deaths have been confirmed globally [35]. The onset of the COVID-19 pandemic has triggered a need for integrated management tools capable of working on a remote basis, thus limiting physical contact among different actors. In this scenario, the different GATEKEEPER Spaces can provide the technological basis to cover the necessities of patients with COVID-19, citizens in quarantine or lockdown, and health and social organizations dealing with the pandemic. RUC9 will tackle this challenge at 2 different levels. At the health care level, several sensing technologies, available in the GATEKEEPER Things Catalogue, can be leveraged and integrated into an unobtrusive mobile data collection platform (ie, based on smartphones, smart trackers, etc), able to monitor key parameters in COVID-19 (ie, heart rate, respiratory rate, and blood oxygen saturation). At the social care level, GATEKEEPER can provide technological solutions dealing with the complexities of social isolation, thus supporting the study population’s self-monitoring at individual and community scale, in synergy with the existing community or social area services.

### Implementation Studies

The establishment of the implementation sites and RUCs is the basis for the definition and execution of large-scale implementation studies. It is key to conceive the different studies individually, with their own target study population, interventions, and evaluation, but also collectively, as a federation of studies aiming to not only test the effectiveness of a given eHealth intervention but also assess the overall performance of a platform hosting the different interventions in a federated way. The promotion of common validated tools measuring activation, adherence, compliance, and outcomes is highly recommended, but it is even more important to consider a common evaluation framework. In this regard, the European Commission promotes the use of indicators for socioeconomic impact assessment by means of the Monitoring and Assessment Framework for the European Innovation Partnership on Active and Healthy Aging [36], which is based on the principles of decision analytic modeling and assesses the impact of health care innovations in terms of health outcomes and resource use. Finally, in the case of GATEKEEPER, this has translated into 25 implementation studies across Europe and Asia targeting up to 50,000 participants.

## Discussion

### Principal Results

This paper describes how the GATEKEEPER consortium tackled the challenge of implementing and testing a platform (embodied in an open source, European, standard-based, interoperable, and secure framework) serving broad study populations of aging citizens with heterogeneous needs, rather than disease-specific eHealth systems or solutions focusing on well-defined subgroups of citizens with homogeneous characteristics. Accordingly, we presented the rationale for the identification and definition of the most appropriate group of settings for the platform to be tested, including (1) the adoption of a double stratification pyramid reflecting at the same time the overall health of the target study population for a given intervention as well as the intensity of the proposed interventions, (2) the identification of a set of principles guiding the selection of implementation countries and sites, and (3) the elaboration of some guidelines for the selection of RUCs so they can be scientifically sound and clinically relevant while covering the whole spectrum of citizen complexities and intervention intensities. Finally, this has been completed with a description of the chosen implementation sites and RUCs, specific aims, and clinical relevance and scientific interest in the GATEKEEPER project.

### Comparison With Existing Literature

In recent years, the European Commission has shifted its scope in relation to eHealth implementation. After years of encouraging eHealth projects tackling a given disease in closed-controlled settings, the focus is moving toward fostering integrated programs and, later on, whole structures capable of sustaining multiple such programs while promoting big data approaches with a secured data flow. Projects, such as CONNECARE [37,38] and ACTIVAGE [9,16] have paved the way for even broader projects, such as GATEKEEPER [10] or Smart4Health [39], where the focus is not on specific health problems but on creating an ecosystem for securely and trustfully sharing all kinds of health-related data and making it accessible to key stakeholders so it can be of a better use for the sake of improving healthy aging and ultimately the health-related quality of life at a population level. Unfortunately, the challenge of assessing such ecosystems remains unexplored terrain.

### Implications for Research and Practice

The creation of a European Data Space (EDS) [40] is one of the priorities of the European Commission for the period of 2019-2025, and the health sector will have a key role in this EDS. Aiming to promote better exchange and access to different types of health data and patient-generated data, the EDS should not only support health care delivery (primary use of data) but also health research and health policy-making purposes (secondary use of data). This new vision encompasses many challenges, including (1) technical requirements, such as the need for supporting interoperability that GATEKEEPER tackles by extending the HL7 Fast Health care Interoperability Resources (standard for the exchange of health care data among the different health organizations and partners; (2) organizational and managerial challenges, such as the need for standardizing the definitions of RUCs and the use of advanced pilot monitoring tools such as Microsoft Power BI dashboards capable of updating in real time; and (3) analytical and evaluation challenges, such as the need for a common evaluation framework that in the case of GATEKEEPER is tackled by using the Monitoring and Assessment Framework for the European Innovation Partnership on Active and Healthy Aging framework. Moreover, real-world implementations mixing data from consumer-grade and medical-grade apps and devices pose challenges on their own, as it may be difficult to guarantee that only medical-grade data will be taken into account for medical decisions. This paper brings an insight into how to tackle key early decisions in the process of assessing complex eHealth platforms and ecosystems. That is, the selection of an optimal group of locations, RUCs, and settings for the multinational large-scale piloting of the platform.

### Conclusions

In conclusion, the approach presented in this paper is leading the current trends and the view of the European Commission, and it provides a methodology for setting up the required large-scale pilots, each with its specific features, that will eventually sustain the creation and development of the EDS. Moreover, this blueprint could be expanded to contexts and constraints outside Europe, as demonstrated by the inclusion of Asian implementation sites in GATEKEEPER.

## Abbreviations

CCM: chronic care model
DM: diabetes mellitus
DALY: disability-adjusted life-year
EDS: European Data Space
EIP on AHA: European Innovation Partnership on Active and Healthy Aging
HF: heart failure
HBP: high blood pressure
KET: key enabling technology
PD: Parkinson disease
RUC: reference use case
WHO: World Health Organization

## References

[1] Kontis V, Bennett JE, Mathers CD, Li G, Foreman K, Ezzati M (2017). Future life expectancy in 35 industrialised countries: projections with a Bayesian model ensemble. Lancet.

[2] Partridge L, Deelen J, Slagboom PE (2018). Facing up to the global challenges of ageing. Nature.

[3] (2020). Decade of healthy ageing: baseline report. World Health Organization.

[4] Eysenbach G (2001). What is e-health?. J Med Internet Res.

[5] Beks H, King O, Clapham R, Alston L, Glenister K, McKinstry C, Quilliam C, Wellwood I, Williams C, Shee AW (2022). Community health programs delivered through information and communications technology in high-income countries: scoping review. J Med Internet Res.

[6] Bernardo J, Apóstolo J, Loureiro R, Santana E, Yaylagul NK, Dantas C, Ventura F, Duque FM, Jøranson N, Zechner M, van Staalduinen W, De Luca V, Illario M, Silva R (2022). eHealth platforms to promote autonomous life and active aging: a scoping review. Int J Environ Res Public Health.

[7] Scheibner J, Sleigh J, Ienca M, Vayena E (2021). Benefits, challenges, and contributors to success for national eHealth systems implementation: a scoping review. J Am Med Inform Assoc.

[8] Tighe SA, Ball K, Kensing F, Kayser L, Rawstorn JC, Maddison R (2020). Toward a digital platform for the self-management of noncommunicable disease: systematic review of platform-like interventions. J Med Internet Res.

[9] Medrano-Gil AM, de los Ríos Pérez S, Fico G, Montalvá Colomer JBM, Sáncez GC, Cabrera-Umpierrez MF, Arredondo Waldmeyer MT (2018). Definition of technological solutions based on the internet of things and smart cities paradigms for active and healthy ageing through cocreation. Wirel Commun Mob Comput.

[10] GATEKEEPER project.

[11] Barceló A, Luciani S, Agurto L, Orduñez P, Tasca R, Sued O (2012). Improving Chronic Illness Care through Integrated Health Service Delivery Networks.

[12] Wallace E, Stuart E, Vaughan N, Bennett K, Fahey T, Smith SM (2014). Risk prediction models to predict emergency hospital admission in community-dwelling adults: a systematic review. Med Care.

[13] (2018). Classification of digital health interventions v1.0: a shared language to describe the uses of digital technology for health. World Health Organization.

[14] (2021). World economic outlook database. International Monetary Fund.

[15] European innovation partnership on active and healthy ageing (EIP on AHA) reference sites. European Commission.

[16] ACTIVAGE Project.

[17] Luxton DD (2015). Artificial Intelligence in Behavioral and Mental Health Care.

[18] Bravo J, Hervás R, Fontecha J, González I (2018). m-Health: lessons learned by m-Experiences. Sensors (Basel).

[19] Ienca M, Vayena E, Blasimme A (2018). Big data and dementia: charting the route ahead for research, ethics, and policy. Front Med (Lausanne).

[20] Lim WS, Canevelli M, Cesari M (2018). Editorial: dementia, frailty and aging. Front Med (Lausanne).

[21] Roski J, Bo-Linn GW, Andrews TA (2014). Creating value in health care through big data: opportunities and policy implications. Health Aff (Millwood).

[22] Clark A, Ng JQ, Morlet N, Semmens JB (2016). Big data and ophthalmic research. Surv Ophthalmol.

[23] Ghani KR, Zheng K, Wei JT, Friedman CP (2014). Harnessing big data for health care and research: are urologists ready?. Eur Urol.

[24] Safiri S, Carson-Chahhoud K, Noori M, Nejadghaderi SA, Sullman MJM, Heris JA, Ansarin K, Mansournia MA, Collins GS, Kolahi AA, Kaufman JS (2022). Burden of chronic obstructive pulmonary disease and its attributable risk factors in 204 countries and territories, 1990-2019: results from the global burden of disease study 2019. BMJ.

[25] Vestbo J, Woodcock A (2017). Clinical trial research in focus: time to reflect on the design of exacerbation trials in COPD. Lancet Respir Med.

[26] Lin X, Xu Y, Pan X, Xu J, Ding Y, Sun X, Song X, Ren Y, Shan PF (2020). Global, regional, and national burden and trend of diabetes in 195 countries and territories: an analysis from 1990 to 2025. Sci Rep.

[27] Bloem BR, Okun MS, Klein C (2021). Parkinson's disease. Lancet.

[28] GBD 2016 Parkinson's Disease Collaborators (2018). Global, regional, and national burden of Parkinson's disease, 1990-2016: a systematic analysis for the Global Burden of Disease Study 2016. Lancet Neurol.

[29] GBD 2019 Diseases and Injuries Collaborators (2020). Global burden of 369 diseases and injuries in 204 countries and territories, 1990-2019: a systematic analysis for the Global Burden of Disease Study 2019. Lancet.

[30] Anand IS, Tang WHW, Greenberg BH, Chakravarthy N, Libbus I, Katra RP (2012). Design and performance of a multisensor heart failure monitoring algorithm: results from the multisensor monitoring in congestive heart failure (MUSIC) study. J Card Fail.

[31] (2021). WeRISE App.

[32] EIP on AHA blueprints. European Commision.

[33] Project MARIO.

[34] Mills KT, Stefanescu A, He J (2020). The global epidemiology of hypertension. Nat Rev Nephrol.

[35] WHO coronavirus (COVID-19) dashboard. World Health Organization.

[36] (2021). Monitoring and assessment framework for the European innovation partnership on active and healthy ageing. European Commision.

[37] Personalised connected care for complex chronic patients. CORDIS.

[38] de Batlle J, Massip M, Vargiu E, Nadal N, Fuentes A, Bravo MO, Miralles F, Barbé F, Torres G (2021). Implementing mobile health-enabled integrated care for complex chronic patients: intervention effectiveness and cost-effectiveness study. JMIR Mhealth Uhealth.

[39] Smart4Health project.

[40] European health data space. European Commision.

